# Apoptotic Death of Cancer Stem Cells for Cancer Therapy

**DOI:** 10.3390/ijms15058335

**Published:** 2014-05-12

**Authors:** Ying-Chun He, Fang-Liang Zhou, Yi Shen, Duan-Fang Liao, Deliang Cao

**Affiliations:** 1 Division of Stem Cell Regulation and Application, College of Medicine, Hunan University of Traditional Chinese Medicine, Changsha 410208, China; E-Mails: yingchunhe@aliyun.com (Y.-C.H.); zhoufangliang305@163.com (F.-L.Z.); 2 Department of Medical Microbiology, Immunology & Cell Biology, Simmons Cancer Institute, Southern Illinois University School of Medicine, 913 N, Rutledge Street, Springfield, IL 62702, USA; E-Mail: yshen@siumed.edu

**Keywords:** cancer stem cells, side population, apoptosis, apoptotic inducers, apoptotic death pathways, and cancer therapy

## Abstract

Cancer stem cells (CSCs) play crucial roles in tumor progression, chemo- and radiotherapy resistance, and recurrence. Recent studies on CSCs have advanced understanding of molecular oncology and development of novel therapeutic strategies. This review article updates the hypothesis and paradigm of CSCs with a focus on major signaling pathways and effectors that regulate CSC apoptosis. Selective CSC apoptotic inducers are introduced and their therapeutic potentials are discussed. These include synthetic and natural compounds, antibodies and recombinant proteins, and oligonucleotides.

## 1. Introduction

Management of patients with advanced malignancies is a worldwide problem. Tumors could be reduced or eliminated through surgical operation, radiotherapy or chemotherapy, but the disease-free survival of patients with advanced cancer is limited thus far. Chemo- and radioresistance widely exists, and recurrence occurs often within six months after primary treatment. Cancer cell dormancy, host immune insult due to tumors or high dose chemotherapy, radiotherapy and surgical operation, *etc*. may lead to treatment failure and tumor recurrence. However, recent studies suggest that the few cells that exist in the tumor and have the characteristics of stem cells may be causative of cancer metastasis, recurrence, and drug resistance. These cells are named cancer stem cells (CSCs) or cancer initiating cells (CICs). Compared to regular tumor cells, unique aberrant gene expression and signaling transduction are recognized in CSCs. This current review discusses the new breakthroughs and discoveries in the CSCs, with a focus on apoptotic signaling pathways and selective inducers of CSC apoptosis.

## 2. Cancer Stem Cells (CSCs)

Early in 1875 Julius Cohnheim introduced a theory that tumors may arise from stem cells left over from embryonic development [1]; but the concept of CSCs was put forwarded for the first time in 1994 [2]. Thereafter; leukemia stem cells were first isolated in 1997 [3]; and CSCs were isolated and characterized from solid tumors in breast cancer in 2003 [4]. From the consensus of an American Association for Cancer Research (AACR) workshop in 2006, CSCs are defined as a kind of cell that possess stem cell-like properties, *i.e.*, self-renewal and pluripotency [5]. CSCs usually account for 1–4 in 100 leukemia cells [6,7] or 1 in 1000–5000 cells in lung; ovarian; and breast cancers [8]; but have strong tumorigenicity; metastaticity and resistance to radio- and chemotherapy; playing critical roles in cancer progression and therapeutic response [9,10].

CSCs are heterogenetic. The CSCs isolated from different stages or grades of the same type of tumors are distinct, while CSCs from the primary and metastatic tumors are different [11,12]. Even in the same tumor, there co-exist different CSC pools, and the distinct CSC subpopulations within a tumor could interconvert. For instance, two molecularly distinct populations of leukemic stem cells (LSCs) co-exist and are hierarchically ordered in primary human CD34(+) acute myeloid leukemia; one LSC population gives rise to the other [13]. CSC heterogeneity also exists in solid tumors. Three cell populations differing in tumorigenicity and self-renewal are identified in estrogen receptor-negative breast tumors [14]. Two highly tumorigenic CSC populations that differ in CD34 expression but are enriched in integrins co-exist at the cancer-stroma interface and display different tumor growth properties [15]. The similar phenomenon is observed in ovarian carcinoma [16], colorectal cancer [17] and PTEN-deficient glioblastoma [18].

Understanding of CSCs has advanced in the past decade. Newer concepts of CSCs consider that CSCs are a “status”, but not a fixed, immutable and frozen cell population. CSCs and non-CSCs exist in a dynamic equilibrium and could interconvert [19,20]. Non-CSCs could acquire the properties and tumor formation ability of CSCs by reprogramming [20,21]. In fact, if CSCs are a fixed cell population, the ratio of CSCs should be progressively reduced with proliferation of cancer cells, but it is clearly not the case. The proportions of CSCs in cancer cell lines remain about 0.1% in the constant culture [10,22]. The microenvironment plays a critical role in CSC division and interconversion. Myofibroblast-secreted factors restore CSC phenotypes in more differentiated colon cancer cells *in vitro* and *in vivo* [23]. Hypoxia-inducible factor (HIF2α) promotes the self-renewal of the stem cells and enhances a more stem-like phenotype in the non-stem population [24–26]. The CSCs may develop *de novo* from differentiated cancer cells (*i.e.*, reprogramming) by the induction of microenvironment. Therefore, the hierarchical model of mammalian CSCs should be considered as bidirectional between stem and non-stem cells of the tumor [20,21].

## 3. Apoptotic Signaling in CSCs

**Figure 1.**
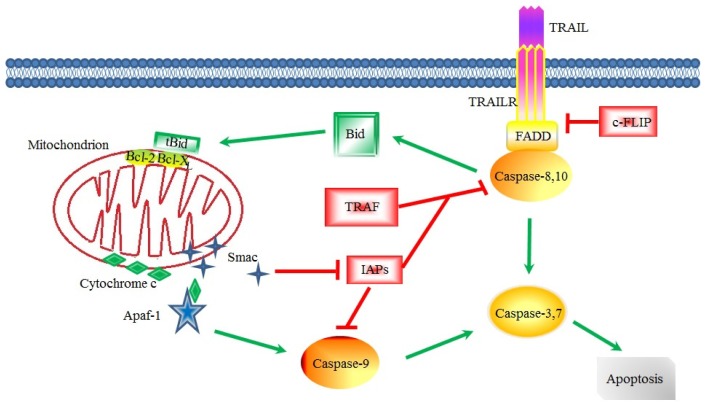
TRAIL-induced extrinsic apoptotic pathway. The apoptotic pathway is activated when the ligand (TRAIL) binds to the death receptor, followed by the recruitment of adaptor molecule FADD and activation of caspase-8 and -10. Activated caspase-8 and -10 activate the caspase-3 leading to apoptosis or cleave Bid to tBid that binds to Bcl-X_L,_ triggering release of mitochondrial cytochrome c and Smac into the cytosol. Assembly of cytochrome c with apoptotic protease-activating factor-1 (Apaf-1) activates caspase-9, which in turn activate caspase-3 and -7, leading to apoptosis. C-FLIP inhibits the activation of caspase-8 by suppressing its combination with FADD. IAPs inhibit caspase-9 or form a complex with TRAF to inhibit caspase-8. Smac can bind to IAPs and attenuate their inhibitory effects on caspase-9.

Apoptosis is an active, strictly regulated, and energy-dependent cell death process [27]. In mammalian cells, apoptosis is regulated via two different pathways, *i.e.*, the extrinsic and intrinsic pathways. Caspases play important roles in apoptosis. The activation of caspase family proteins triggered by these two signaling pathways results in a series of cellular substrate excision and changes, such as chromatin condensation, DNA fragmentation, membrane blebbing, and cell shrinkage [28]. The extrinsic pathway is triggered through the binding of extracellular proapoptotic ligands to cell surface receptors, known as death receptors, such as CD95, nerve growth factor receptor (NGFR), and TNF-related apoptosis-inducing ligand (TRAIL) receptors (Figure 1) [29,30]. After binding to the receptor, a death-inducing signaling complex (DISC) composed of the Fas associated death domain (FADD) and procaspase-8 and -10 is formed [31–33], and this protein complex activates procaspase-8 and -10 inside itself, and then cleaves procaspase-3 and initiates the apoptosis process [34]. In the extrinsic pathway, the downregulation of cellular FLICE inhibitory protein long isoform (c-FLIPL) by ubiquitination at lysine residue (K) 195 occurs [35]. The intrinsic pathway, also known as the mitochondrial pathway, is induced by a variety of stress signals that trigger cellular and DNA damage, such as ionizing radiation, cytotoxic agents, and growth factor withdrawal. They lead to mitochondrial outer membrane permeabilization (MOMP) and transcription or post-translational activation of BH3-only proapoptotic B-cell leukemia/lymphoma 2 (Bcl-2) family proteins [29]. The mitochondrial permeability is a key step in the apoptosis cascade and mediated by Bcl-2 family proteins. The mitochondrial permeability allows the release of apoptotic proteins, such as cytochrome c and second mitochondria-derived activator of caspase (Smac), from the intermembrane space into cytosol [36,37]. The assembly of cytochrome c and apoptotic protease-activating factor-1 (Apaf-1) activates caspase-9 which in turn activates the effectors caspase-3, -6, and -7, leading to apoptosis [29]. Inhibitors of apoptosis protein (IAP) prevent both intrinsic and extrinsic apoptosis by inhibiting caspase activity, which represents the last protective measure against apoptosis [38]. Death signaling can also be activated by c-Jun *N*-terminal kinase (JNK) signaling which leads to phosphorylation of Bcl-xL at Ser62, decreasing its anti-apoptotic activity in the intrinsic pathway [35]. Intrinsic and extrinsic apoptosis pathways are both disordered in cancer cells; and apoptosis evasion is one of the hallmarks in cancer cells [39,40].

Apoptotic signaling pathways, including extrinsic and intrinsic pathways, are also deregulated in CSCs. In glioblastoma and lung CSCs, the death receptors (DR) mediating the extrinsic pathway are expressed at a high level [41], and the upregulation of DR4 in colon CSCs leads to chemo-resistance [42]. The FLICE-like inhibitory proteins (cFLIP) are a negative modulator of death receptor-induced apoptosis, consisting of two subtypes: long cFLIP (cFLIPL) and short cFLIP (cFLIPS) [43,44]. In CD133+ glioblastoma, breast cancer, and T-cell acute leukemia cells, the cFLIPs are upregulated [45,46]. Silencing of cFLIPs by siRNA restores cell sensitivity to death stimuli, suppressing CSC self-renewal and tumor metastasis [47,48]. It was reported that insufficient expression of death receptors and upexpression of c-FLIPs leads to CSCs-enriched neurosphere resistance to TRAIL [49].

Survivin is an anti-apoptotic protein, belonging to the inhibitors of apoptosis protein (IAP) family that regulates cell division, apoptosis and pluripotency [38,50,51]. Survivin is enriched in hematopoietic stem cells, neuronal precursor cells, CD34(+)/38(−) AML stem cells and glioblastoma and astrocytoma CSCs [52–54]. Other IAP proteins upregulated in CSCs include XIAP, c-IAP1, and Livin [45,54].

Dysregulation of the intrinsic pathway in CSCs is mainly reflected in Bcl-2 family proteins and the DNA damage response. Bcl-2 family proteins are composed of anti-apoptotic proteins (Bcl-2, Bcl-X_L_ and Mcl-1) and pro-apoptotic molecules (Bax, Bak, Bid, Bim, Bik, Noxa and Puma [55]. It is the imbalance of anti- to pro-apoptotic protein ratio rather than a specific molecule expression level that tips the balance to cell survival and regulates sensitivity to apoptotic stimuli [55]. In most tumors, anti-apoptotic Bcl-2 family proteins are overexpressed in CSCs [56]. For instance, CD133+ glioma CSCs express a high level of anti-apoptotic proteins Bcl-2 and Bcl-X_L_ [45,57], and high expression of Mcl-1 correlates with resistance to the Bcl-2 inhibitor ABT-737 in glioma CSCs [57]. In colon CSCs, Bcl-2 is increased and inhibits apoptosis and autophagy [58]. Downregulation of Bcl-2 or upregulation of Bax induces apoptosis of CSCs [37,59]. Therefore, inhibition of the mitochondrial death cascade has been attractive for CSC-targeted therapeutic intervention of cancers.

DNA damage response (DDR) is tumor suppressor p53-mediated cell-cycle arrest, DNA repair, and apoptosis in response to DNA damage [60]. Glioma CSCs isolated from human glioma xenografts and primary glioblastoma produce radio-resistance by preferential activation of the DNA damage response, and the radio-resistance of CSCs could be reversed by specific inhibition of Chk1 and Chk2 checkpoint kinases, upstream activators of p53 [61]. In addition, nuclear factor-kappa B (NF-κB) signaling regulates apoptosis of CSCs through affecting the expression of pro and anti-apoptotic proteins. In leukemic stem cells, NF-κB is activated [62] and increases the quiescent LSC number [63]. Breast CSCs exhibit sensitivity and apoptosis to NF-κB pathway inhibitors, such as parthenolide, pyrrolidinedithiocarbamate, and diethyldithiocarbamate, and the expression of CD24 in CD44+ breast CSCs potentiates DNA damage-induced apoptosis by suppressing NF-κB signaling [64–66].

MiRNAs are small non-coding RNAs (ncRNAs) that regulate protein translation by binding to target mRNAs [67]. MiRNAs are widely involved in cancer cell growth, migration, invasion, and drug sensitivity [68,69]. In tumor cells, miRNA expression is dysregulated, and function as tumor suppressors or oncogenes. For instance, miR-223, miR-122, and miR-26 function as tumor suppressors of liver carcinogenesis whereas miR-130b, miR-221, and miR-222 are oncogenic factors [70–74]. Emerging evidence indicates that miRNAs are key regulators of stemness. For instance, miRNAs let-7, miR-21, miR-22, miR-34, miR-101, miR-146a, and miR-200 affect CSC phenotypes and functions through targeting oncogenic signaling pathways [75]. Other miRNAs appear to affect the fate of CSCs by controlling their self-renewal. For example, MiR-34 inhibits human pancreatic CSCs by regulating Notch and bcl-2 gene expression [76], and miRNA-34a suppresses glioma CSC growth by targeting several oncogenes [77]. There is also a difference in miRNA expression levels between CSCs and non-CSC cancer cells [78]. For example, mir-21 and mir-302 expression is increased in CSCs whereas let-7a is downregulated. In addition, mir-372, mir-373, and mir-520c-5p are expressed at higher levels in non-CSC than in CSCs [78].

## 4. Apoptotic Inducers of CSCs

The death evasion of CSCs accounts for failure of existing therapies to eradicate tumors [79,80]. Increasing recognition of signal effectors of apoptosis pathways in CSCs paves the way for the development of more specific inducers targeting key signaling components [81,82]. To date, a variety of selectively CSC-targeting agents have been developed, and they are classified as natural compounds (e.g., traditional Chinese herb extracts and antibiotics), synthetic chemicals, antibodies or recombinant proteins, and oligonucleotides.

### 4.1. Natural Compounds

#### 4.1.1. Natural Compounds from Traditional Chinese Medicines

Genistein (a prominent isoflavone) inhibits cell growth and induce apoptosis by suppressing the Notch signaling pathway [83]. Soy isoflavone genistein and blueberry polyphenolic acids repress mammosphere formation of breast cancer cells [84,85], and 20 (s)-ginsenoside Rg3 inhibits proliferation of colon CSCs and induces apoptosis through caspase-9 and caspase-3 pathways [86]. NV-128 is an isoflavone derivative that targets mitochondria of CD44+/MyD88+ ovarian CSCs and induce apoptosis by promoting a status of cellular starvation, which activates two independent pathways: the AMPKα1 pathway that causes mTOR inhibition and the mitochondrial MAP/ERK pathway that leads to loss of mitochondrial membrane potential [87]. Broussoflavonol B, a chemical purified from the bark of the Paper Mulberry tree (broussonetia papyrifera), downregulates estrogen receptor (ER)-α36 expression and inhibits growth of ER-negative breast cancer stem-like cells and induces apoptotic cell death [88]. Shikonin and topotecan, as topoisomerase I inhibitors, can induce apoptosis and inhibit growth of glioma cells and glioma stem cells [89]. Curcumin can induce CD133+ rectal CSC apoptosis and significantly increase radiosensitivity of CSCs [90]. Curcumin and piperine, alone or in combination, can suppress breast CSC growth [91]. In addition, resveratrol, a natural polyphenolic compound, inhibits the growth of breast CSCs and induces apoptosis through upregulation of DAPK2 and BNIP3 and downregulation of fatty acid synthetase (FAS) [92]. Furthermore, morusin induces apoptosis of cervical CSCs by downregulating NF-κB/p65 and Bcl-2 and upregulating Bax and caspase-3 in a dose-dependent manner [59]. Table 1 summarizes these natural compounds.

#### 4.1.2. Antibiotics

Salinomycin (a polyether) selectively kills breast CSCs 100 times more effectively than anti-cancer agent paclitaxel [93]. Salinomycin triggers apoptosis of CSCs through multiple mechanisms, such as increasing the expression of death receptor-5 (DR5), caspase-8, and FADD, decreasing expression of FLIP, and activating caspase-3 and poly ADP-ribose polymerase (PARP) cleavage [94].

### 4.2. Synthetic Compounds

As the prospective feature of CSC apoptosis in cancer therapy, a variety of small chemicals have been chemically synthesized and tested. Fenretinide is a synthetic retinoid developed by Ortho-McNeil Company and the United States National Cancer Institute. This chemical selectively inhibits colony formation of CD34+ AML cells, but has no effect on normal CD34+ cells; fenretinide also reduces the *in vivo* engraftment of AML CD34+ cells, but has no effect on normal hematopoietic stem cells in non-obese diabetic SCID mice [95]. In addition, a dopamine antagonist thioridazine can selectively destroy LSCs, but not normal hematopoietic stem cells [96].

Aspirin inhibits CSCs by decreasing the expression of Lgr 5 protein via both COX-2 dependent and independent pathways, and contributes to the prevention and treatment of colorectal cancer [97]. IMD-0354, an inhibitor of NF-κB, inhibits phosphorylation of IκBα and release of NF-κB proteins, and thus induces breast CSC apoptosis [98]. LDE225 (also named NVP-LDE-225 or Erismodegib), is a novel specific Smoothened antagonist and Hedgehog signaling pathway inhibitor. This chemical suppresses the growth and spheroid formation of prostate CSCs and induces apoptosis by affecting the expression of multiple pro-and anti-apoptotic proteins; LDE225 also stimulates Gli-DNA interaction and transcriptional activity [99].

Survivin has been an effective target for the inhibition of CSC proliferation. For instance, PF-03084014 could suppress the expression of survivin and MCL1 and diminish CSCs in triple-negative breast cancer tumor models [100], and FH535 (*N*-(2-Methyl-4-nitrophenyl)-2,5-dichlorobenzene-sulfonamide) and sorafenib inhibit liver CSC growth and proliferation by targeting survivin [101]. In addition, STX-0119, an inhibitor of signal transducer and activator of transcription (STAT) 3, inhibits the expression of STAT3 target genes, such as survivin and c-Myc and induces CSC apoptosis [102].

### 4.3. Antibodies and Recombinant Proteins

Several recombinant TRAIL receptor agonists and IAPs are being implemented thus far in phase I and II clinical trials, such as the 2/TNF-related apoptosis-inducing ligand (Apo2L/TRAIL) that targets death receptors and induces selective apoptosis of CSCs [103]. Bevacizumab is a recombinant humanized monoclonal antibody that targets vascular endothelial growth factor (VEGF) and suppresses angiogenesis in tumors, leading to collapse of the CSC niche. Microvessel density and tumor growth and CD133+/nestin CSCs are decreased in U87 glioma xenografts treated with bevacizumab in nude mice [104,105]. In addition, IL-4 protects the tumorigenic CD133+ CSCs in human colon carcinoma from apoptosis, and the anti-IL-4 antibody or IL-4R alpha antagonists induces apoptosis of CSCs and markedly sensitizes them to chemotherapeutic drugs [106]. Antibodies against CD47, which is expressed at a high level in ALL, can also effectively kill leukemia stem cells [107].

### 4.4. Oligonucleotides

Mature microRNAs (miRNAs) at 18–25 nucleotides in length are produced from longer primary miRNA (pri-miRNA) transcripts through sequential processing by RNase Drosha and Dicer1 [108,109]. MiRNAs negatively regulate the expression of targeted mRNAs involved in stem cell self-renewal, proliferation, differentiation, and apoptosis [110]. MiRNAs may exert anti- or pro-apoptotic effect depending on the targeted mRNAs [111,112], thus being selectively targeted in order to trigger apoptosis of CSCs for cancer therapy.

Stranded antisense oligonucleotides (AS-ODN) are synthetic short chain DNA at 12–30 nt in length, complementary to a particular mRNA strand. An AS-ODN hybridizes with the targeted mRNA through Watson-Crick base pairing, and thus blocks translation of the targeted gene and inhibits its role. In human lung adenocarcinoma cells, an AS-ODN targeting survivin decreases its protein level in a dose-dependent manner and leads to apoptosis and chemotherapeutic sensitivity. The XIAP AS-ODN effectively induces apoptosis and increases the sensitivity of tumor cells to Taxol, etoposide, and doxorubicin [113,114]. Successful CSC-targeting of oligonucleotides was reported in an approach to telomerase. The telomere and telomerase play essential roles in the regulation of the lifespan of human cells. Imetelstat sodium (GRN163) is a 13-mer oligonucleotide N3′–P5′ thiophosphoramidate (NPS oligonucleotide) covalently attached to a C16 (palmitoyl) lipid moiety. GRN163 targets the active site of telomerase, competitively inhibiting its enzymatic activity. The Marian group [115] reported that Imetelstat reduces brain glioma CSCs telomere length, inhibits their proliferation, and ultimately induces apoptosis.

### 4.5. Combined Application of Apoptotic Inducers

Apoptotic inducers show potential pro-apoptotic effects in CSCs. However, CSCs have complex etiology and pathogenesis, characterized with considerable crosstalk and redundant signaling pathway networks. Targeting a single molecule or pathway may have limited efficacy in cancer therapy. Therefore, scientists use approaches combining applications of apoptotic inducers to improve therapeutic efficacy.

Lapatinib is a small synthetic, dual tyrosine kinase inhibitor of epidermal growth factor receptor (EGFR) and human epidermal growth factor receptor type 2 (HER2). Lapatinib can significantly improve the sensitivity of CSCs to chemotheraputic drugs in adjuvant chemotherapy [116]. Combination of methylene blue (a P-gp inhibitor) with doxorubicin enhances tumor cell apoptosis and suppresses tumor growth, significantly improving survival of BALB/c mice bearing syngeneic JC adenocarcinoma [117]. Vinorelbine (a semi-synthetic derivative of vinblastine) stealth liposomes and parthenolide are developed to eradicate cancer cells [118]. The parthenolide significantly enhances the cytotoxicity of vinorelbine in MCF-7 CSCs [118].

Doxorubicin is a DNA-toxic antitumor agent. Metformin, an agent for diabetes, can inhibit cell transformation and selectively kill CSCs in breast cancer [119]. Metformin combined with doxorubicin can kill both CSCs, reduce tumor masses, and prevent metastasis and recurrence much more effectively than either agent alone [119]. In addition, it is also reported that Tamoxifen (a synthetic estradiol competitive antagonist) can enhance breast CSCs sensitivity to daunorubicin, demonstrating a synergistic effect [120]. Furthermore, a combination of salinomycin with gemcitabine eliminates the engraftments of human pancreatic cancer more effectively than the individual agent alone [121].

Ectopic expression of miR-128 sensitizes breast CSCs to DNA-damage and proapoptotic effects of doxorubicin [122] whereas miR-145 combined with cationic polyurethane-short branch PEI inhibits the glioblastoma CSC growth and increases their sensitivity to radiotherapy and temozolomide [123].

Combinatory approaches were also tested in synthetic chemicals with antibodies, recombinant proteins and oligonucleotides. For instance, perifosine and TRAIL synergistically activate caspase-8, induce apoptosis, and negatively affect the clonogenic activity of CD34(+) AML cells, but not CD34(+) cells from healthy donors [124]. CD133+ populations in T cell acute leukemia cell line Jurkat and breast cancer cell line MCF7 express high levels of apoptosis inhibitor, c-FLIP, and lead to TRAIL resistance. In these two cell lines suppression of c-FLIP with siRNA sensitizes CSCs to TRAIL and selectively removes CSCs [48].

## 5. Prospects

CSC differentiation is reversible, *i.e.*, the mature tumor cells or precursor cells can obtain CSC properties by dedifferentiation. Understanding of CSCs has explained the heterogeneity, metastasis, recurrence and chemo-/radiotherapy resistance of tumors, and the evasion of apoptosis of CSCs is considered a main mechanism of recurrence and chemoresistance of cancers, representing novel therapeutic potentials. The apoptosis is mediated by complex networks composed of many death and survival signaling molecules and pathways. Manipulating the apoptotic machinery, including activation of pro-apoptotic pathways and inactivation of anti-apoptotic pathways to eradicate CSCs, displays great potentials. Selective induction of CSC differentiation, suppression of their self-renewal, or triggering of their apoptosis by targeting key signaling molecules and microenvironment factors, are certainly attractive explorations to cancer researchers. Combined applications of agents targeting CSC apoptosis are an important advantage improving their antitumor efficacy.

## Figures and Tables

**Table 1.** Natural inhibitors of cancer stem cells (CSCs).

Natural compound	Sources	Tumor types	Ref.
Genistein	Soy	Pancreatic CSCs	[83]
Genistein	Soy	Breast CSCs	[84,85]
Blueberry Polyphenolic Acids	Blueberry	Breast CSCs	[84]
20(*S*)-Ginsenoside Rg3	Panax Ginsen	Colon CSCs	[86]
Nv-128	Soy (Isoflavone Derivative)	Ovarian CSCs	[87]
Broussoflavonol B	Broussonetia Papyrifera	Breast CSCs	[88]
Shikonin	Arnebia Euchroma	Glioma CSCs	[89]
Curcumin	Curcuma Longa L.	Rectal CSCs, Breast CSCs	[90,91]
Piperine	Pepper	Breast CSCs	[91]
Resveratrol	Grape, Peanut, Polygonum Cuspidatum	Breast CSCs	[92]
Morusin	Morus Alba L.	Cervical CSCs	[59]
